# INSIGHTS ON OLDER ADULTS WITH COGNITIVE IMPAIRMENT AND HEARING LOSS: EXPERT PERSPECTIVES

**DOI:** 10.1093/geroni/igae098.2000

**Published:** 2024-12-31

**Authors:** Marcela Blinka, Esther Oh, Peter Hope, Paul Lee, Xueting Tu, Natalie wang, Sevil Yasar, Carrie Nieman

**Affiliations:** Johns Hopkins University, Baltimore, Maryland, United States; Johns Hopkins University, Baltimore, Maryland, United States; Johns Hopkins University School of Medicine, Baltimore, Maryland, United States; Johns Hopkins University, Baltimore, Maryland, United States; Johns Hopkins University, Baltimore, Maryland, United States; Johns Hopkins University, Baltimore, Maryland, United States; Johns Hopkins University School of Medicine, Baltimore, Maryland, United States; Johns Hopkins University School of Medicine, Baltimore, Maryland, United States

## Abstract

Although hearing loss is highly prevalent among older adults, especially among older adults aging with cognitive impairment, few use hearing aids. Older adults with co-morbid cognitive impairment and hearing loss experience multiple barriers to hearing care, including hearing loss often going unrecognized by clinicians and deprioritizing hearing care among competing health priorities. To understand the barriers and facilitators to hearing care among persons living with dementia and inform the development of a hearing care intervention, we conducted semi-structured interviews with 10 experts. Experts included professionals with expertise in dementia, hearing loss, neuropsychiatric symptoms, behavioral intervention development, implementation science, and technology adoption. We used purposive sampling and snowball recruitment to recruit experts with demonstrated experience and diverse backgrounds. Qualitative content analysis was used to identify themes. Among the experts, 56% self-identified as male, and 78% self-identified as white. Experts highlighted the importance of a multi-component approach to hearing care, including education on hearing and dementia, management of neuropsychiatric symptoms, and employing effective communication strategies. Experts prioritized the need for interdisciplinary collaboration and dedicated training for clinicians. Experts emphasized the need to engage stakeholders throughout intervention development and testing, including the key role care partners play in prioritizing hearing care and supporting the ongoing use of assistive technologies. Experts also identified the need to integrate practical strategies to support communication needs and hearing technology that is affordable. Our findings support the importance of hearing care within dementia care and identify tangible opportunities to support hearing health among persons aging with cognitive impairment.

